# The pharmaceuticalisation of life? A fictional case report of insomnia with a thought experiment

**DOI:** 10.1186/s13010-021-00109-7

**Published:** 2021-10-02

**Authors:** Emmanuel Bäckryd

**Affiliations:** Pain and Rehabilitation Centre, and Department of Health, Medicine and Caring Sciences, Linköping University, Linköping, Sweden

**Keywords:** Sleep, Sleeplessness, Medicalisation, Pharmaceuticalisation, Hypnotics, Moberg

## Abstract

**Background:**

The safety of sleeping pills has increased dramatically during the last 100 years, from barbiturates to bensodiazepines to modern day so-called Z-drugs.

**Methods:**

The circumstances of prescribing sleeping pills in the early 20^th^ century are illustrated by summarizing the main storyline of a novel by Swedish writer Vilhelm Moberg. This is followed by a thought experiment and a theoretical discussion.

**Results:**

In his 1937 novel *Sömnlös* (Swedish for sleepless) Vilhelm Moberg portrayed existential and relational distress in relation to insomnia. In a thought experiment, past progresses in sleeping pills safety are projected into the future. Thereby, it is claimed that important issues in the area of philosophy of medicine come to the fore. This leads to a theoretical discussion about broader questions concerning the role of the physician, the goals of medicine (as described by Lennart Nordenfelt), the concept of pharmaceuticalisation (as described notably by sociologist of sleep Simon J. Williams and co-workers), and health enhancement (c.f. Carl Elliott and the alleged wish to be better than well).

**Conclusion:**

Insomnia is a prism through which important philosophical and sociological questions related to the practice of medicine can be asked.

## Introduction: the complexity of sleeplessness

According to data available from the open statistical database of the Swedish Board for Health and Welfare, about 10 % of adults in Sweden are prescribed sleeping pills (Anatomical Therapeutic Chemical classification system code N05C) at least once per year [27, 28]. Hence, sleep disturbances are very common. Indeed, the prevalence of insomnia disorder in the general population has been said to be approximately 10-20 % [4].

There is an association between insomnia and various medical and psychiatric conditions [3]. To take an obvious common-sense example, if you get cancer and as a result of that become existentially anxious at the prospect of death, you might have trouble sleeping. Also, many patients with psychiatric disorders complain of sleep disturbances, the relationship often being viewed as “bidirectional” [31]. The interrelationship of insomnia with other medical conditions is informatively summarized by Buysse:

Insomnia disorders have also been categorized as primary and secondary, depending on whether the sleep problem is judged to be caused by another medical or mental disorder or medication/substance use. In practice, it is often difficult to determine whether a concurrent condition actually causes insomnia. Furthermore, insomnia is a risk factor for many of the disorders with which it coexists, including coronary heart disease and depression. For these reasons, the term *comorbid insomnia* has been recommended as preferable to *secondary insomnia*. Although sleep medicine specialists have defined subtypes of primary insomnia, the reliability and validity of primary insomnia and its subtypes are modest at best. […] A separate insomnia diagnosis is not needed for all patients with medical, psychiatric, or other sleep disorders who have insomnia symptoms, and should be made only if the symptoms are severe or constitute an independent focus of clinical attention [4].

Of course, sleep and insomnia can be studied from many different perspectives, e.g. by scholars in humanities and social sciences. Notably, as described by Ekirch, the pattern of sleep in pre-industrial Western societies seems to have been “bimodal” [9, 10], meaning that “typically, after retiring to bed between 9 and 10 p.m., pre-industrial families experienced two major intervals of nocturnal sleep, ‘first sleep’ and ‘second sleep’, bridged shortly past midnight by up to an hour or so of wakefulness in which individuals meditated, made love and conversed” [9]. Hence, it seems that noctural awakenings have not always been viewed as abnormal. Indeed, the field of sociology of sleep can help us problematize conceptions of “normal” sleep [30].

**Fig. 1:**
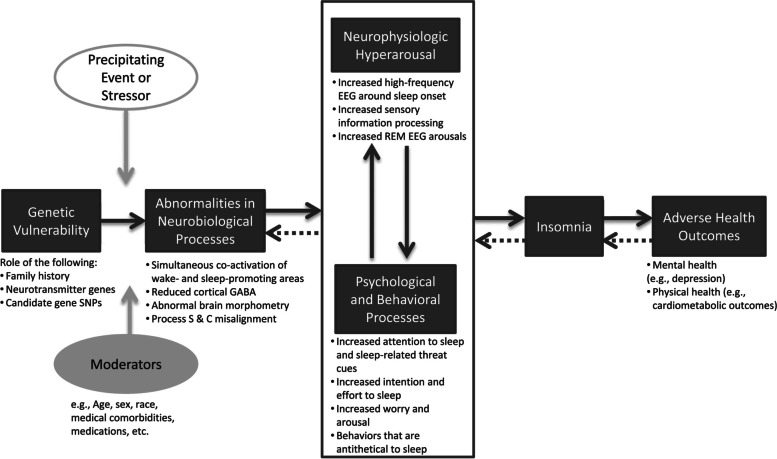
A model of the pathophysiology of insomnia according to Levenson and co-workers [18]. Reused with permission

Going back to the point of view of medical science, a recent review by Levenson and co-workers has summarized the pathophysiology of insomnia, using seven “levels of analysis” [18]. These levels are different perspectives from which insomnia in an individual patient can be viewed. The seven levels are: (1) genetic, (2) molecular, (3) cellular, (4) neuroanatomic/neural circuitry, (5) physiologic, (6) behavioural, and (7) self-report. As can be seen, there is a clear bottom-up hierarchy, from genes at the bottom to behaviour and self-report at the top. The complexity in the model is summarized in Fig. 1.

Hence, insomnia is a multi-faceted problem. In the present paper, a fictional case of insomnia as described by Swedish novelist Vilhelm Moberg will be used as a backdrop for a thought experiment. The paper will unfold in four parts. First, I will very briefly summarize the main storyline of the first part of Moberg’s novel *Sömnlös* (Swedish for sleepless), and existential and relational distress as portrayed in *Sömnlös* will be related to the problem of insomnia. Second, based on Moberg’s portrayal, a thought experiment will be carried out which, third, will lead me to discuss the broader questions of the role of the physician and the goals of medicine, and, fourth, to a discussion of the concept of pharmaceuticalisation. Hence, insomnia is the prism through which more general questions will be asked about the aims and limits of medicine.

## A fictional case report of insomnia

Vilhelm Moberg (1898-1973) is one of the giants of Swedish literature [12]. He is best-known for *The Emigrants* series, a tetralogy published between 1949 and 1959 that describes the fate of a group of emigrants who leave Sweden to seek a better life in the United States. S*ömnlös* is one of Moberg’s earliest novels, published in 1937. To the best of my knowledge, it has never been translated into English. All quotes are my own translation.

The protagonist in *Sömnlös*, Knut Toring, was born in the poor rural province of Småland, but in his youth he left the small village of Lidalycke for a life in Stockholm, the capital city. Toring, now 36 years old, is the reasonably successful editor of a literary magazine that publishes short stories. He is married to Aina and they have two children. Outwardly everything seems fine. But Toring has for a while suffered from sleeplessness. When the novel starts, he has just visited a renowned neurologist who, after a thorough medical examination, has concluded that Toring is completely healthy. There is nothing pathological to be found. This is a simple case of overexertion, the physician tells him, and he gives Toring some practical advice concerning how to proceed when getting to bed. He does not prescribe any sleeping pills because (as he puts it) nobody dies from nervous sleeplessness whereas, on the contrary, it is very possible to poison yourself with sleeping pills.

Toring completely accepts what the neurologist has said and he tries to do as he has been told. But sleep refuses to come. Eventually, at the end of part one of the book (the part which this paper will focus on), Toring quits work and leaves his family, returning to his native village in Småland, first to live with his parents and then on his own on a little farm.

According to Toring’s physician, and using the traditional distinctions outlined in Table 1, Toring, although feeling ill, does not suffer from a disease. This is quite obvious and not especially interesting in itself. What is more interesting is the literary description of how Toring’s fight with insomnia is paralleled by an increasing sense of discontentment with his life in general and work in particular, and with the materialism and pursuit of comfortableness that he associates with city life in Stockholm.

**Table 1:** The three traditional concepts of *disease*, *illness*, and *sickness* [1, 17, 20]

Disease:	Professional perspective	Objective view	What health care personnel think
Illness:	Personal perspective	Subjective view	How it feels for the patient
Sickness:	Social perspective	Inter-subjective	Sick role in a group, community, or society

During his sleepless nights, Toring begins to feel “the presence of the concept of Eternity” ([22], p. 13). He tries to pull himself together; it is no good busying oneself with something that deviates from “a rational way of life” ([22], p. 15). He should focus on the real and factual, on what could be examined and weighed! So why can’t he do that? As Toring lies in his bed, he recollects that, for a few years now, he has not been enjoying his work. He has however somehow managed to control his discomfort by an attitude of resignation. There is nothing to do about it. He has actually been rather successful in holding this feeling of frustration at bay. But instead, insomnia has set in. It is as if the one has given way to the other. And now, after having struggled with sleeplessness for a while, his discontent about work comes back to the fore. In a discussion with his wife, the truth finally bursts forth: “It’s just that I hate my work. … It feels like work kills my soul! … That’s why I want to go back!” ([22], p. 32).

His existential distress has another side that is intertwined with his discontent at work: his discontent with what he considers to be the shallow materialism of city life in Stockholm. His brother-in-law Fredrik is a symbol for this. Fredrik and his wife Berta “lived prudently and rationally” ([22], p. 25). Fredrik had at an early age made up a plan for his life, and this plan is now largely realized: getting a nice job and a family; buying a house with all the comforts of modernity. But Toring does not accept this way of life any longer. In another discussion with his wife, he recuses the Trinity of yearly income, standard of living, and comfort. These three “gods” are enslaving forces that have made the human soul captive. He has sold out his soul for a salary of 10,000 crowns per year, but the living soul inside him can no longer accept this. He can no longer stand “the spirit of materialism” ([22], p. 142).

Hence, Toring feels existentially disconnected, especially concerning work and the materialistic society that he fills encircled by. But his distress is also relational. He has never really connected with anybody in Stockholm: “Outside his family, he did not have any sense of community with people“ ([22], p. 41). Why such loneliness? Even he and his wife Aina are now “outside of each other” ([22], p.81), and finally his marriage breaks down. He quits his job, leaves his family, and moves back to Lidalycke.^1^

## A thought experiment

**Fig. 2:**
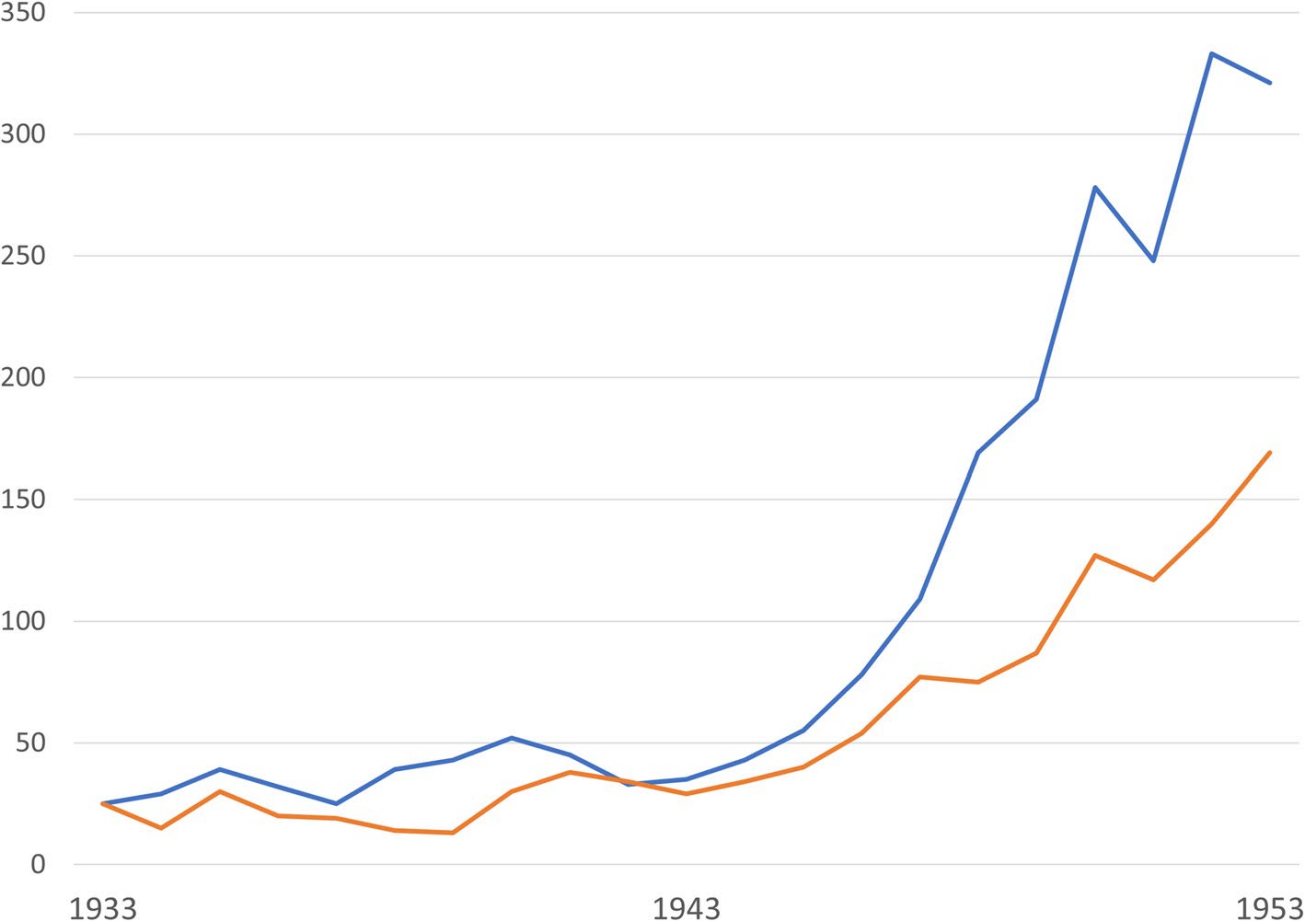
Number of deaths from barbiturate poisoning in England and Wales 1933-1953, according to Registrar-General's Statistical Review. The figure is based on a data table retrieved from [14]. Blue line: Deaths by suicide. Red line: Deaths by accident

This case report may be fictional, but it is not unrealistic. Using the terminology introduced above, Toring suffers from *primary* insomnia and his physician refuses to treat this pharmacologically. The reason given is that of safety, as sleeping pills are dangerous (you can poison yourself with them). Moberg’s book was published in 1937, and at that time practically the only drugs used as sleeping pills were barbiturates; indeed, it was during the 1930s and 1940s that barbiturates attained their greatest popularity and were most widely used [19]. Therefore, Moberg probably refers to barbiturates and he seems to be very up to date concerning their danger, a danger that is illustrated in Fig. 2. The clinical introduction of the much safer benzodiazepines at the end of the 1950s introduced the benzodiazepine era of sleeping pills [19], which itself was succeeded by the advent of the still safer so-called Z-drugs (mainly zopiclone and zolpidem) in the 1990s [26]. Hence, there is a clear progress over time towards safer sleeping pills: from barbiturates to bensodiazepines to Z-drugs. In the following thought experiment, this century-long development towards safer sleeping pills will be projected into the future.

**Fig. 3:**
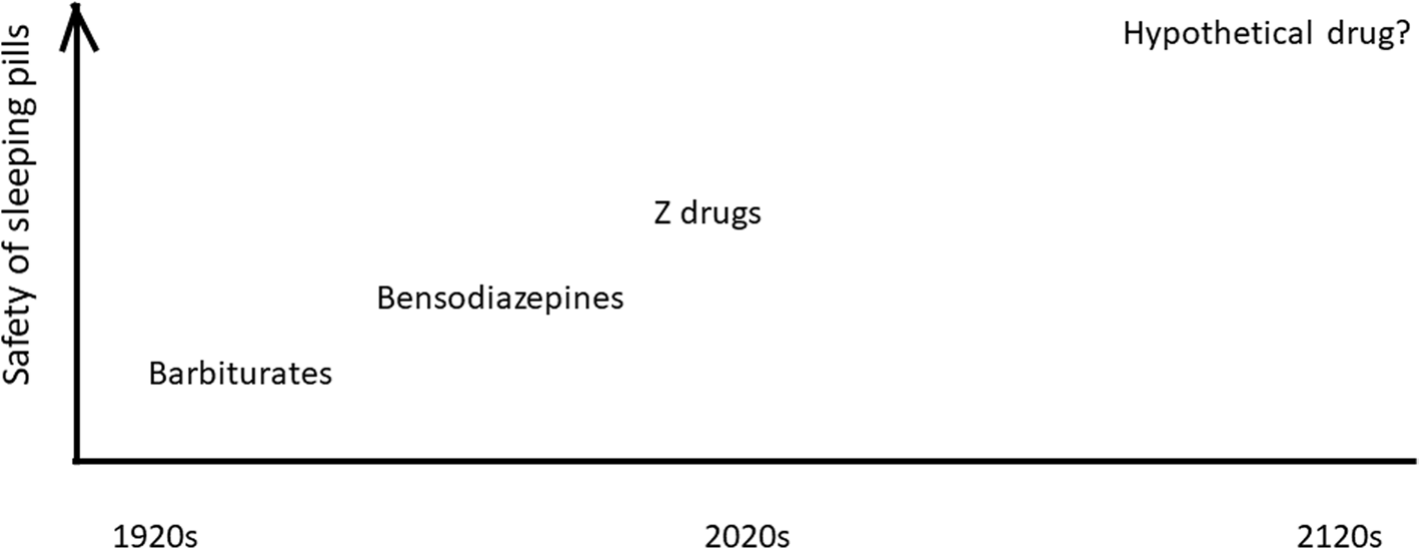
Increasing safety of sleeping pills over time – a thought experiment

What if, in 100 years’ time, sleeping pills are much safer than they are today? Let us in the following thought experiment assume the same situation as the one described by Moberg, i.e. insomnia associated with existential and relational distress, but let us transpose this situation 100 years from now, to year 2120, and let us assume two crucial differences in that hypothetical future: (a) the ability to use neuroimaging biomarkers to precisely pinpoint the neurobiological mechanisms involved in each case of insomnia, and (b) the ability to use that knowledge to prescribe sleeping pills that are both very effective and very safe. The pharmacological side of the thought experiment is summarized in Fig. 3.

This may feel more like science fiction than science but given the medicotechnological advances made during the last 100 years, one should perhaps not be too quick to discard such thoughts. Be that as it may, I think this thought experiment helps us to see the issues at stake more clearly, namely: if a very effective and safe sleeping pill was available, should a doctor prescribe it under the circumstances depicted by Moberg? Remember that Toring’s problems, as portrayed by Moberg , are of an existential and relational nature. Of course, everything Toring experiences, including his insomnia, is realized neurobiologically in the brain, but this is not the question here. The question is: Should a future doctor, under the circumstances portrayed by Moberg, prescribe a sleeping pill if the dangers inherent in such a prescription have been more or less cancelled by the advancement of science?^2^

## The goals of medicine

Becoming a physician is not only about learning about human biology, it is about learning a profession. The scope and extent of this profession are not self-evident and potentially subject to change, and it is therefore interesting to reflect on the goals and limits of medicine as well as on the relation to the so-called pharmaceuticalisation of society. These issues will now be discussed from the point of view of the above-mentioned thought experiment.

How should the goals of medicine be understood? According to Brülde [2], “medical enterprise… [has] a multidimensional goal structure rather than a single goal”, and Brülde argues there are seven irreducible goals, five of them being means to the remaining two (which are ends in themselves). The two ends in themselves are a long life and a high quality of life. This is hardly in itself a revolutionary thought. In an answer to Brülde that moves the discussion forward, one of the things Nordenfelt discusses is different notions of medicine [25]. Under the umbrella of what he calls “health enhancement”, Nordenfelt distinguishes between:

- Medical care
- Rehabilitation
- Nursing
- (Clinical) social care
- Medical prevention
- Health education
- Legal health protection
- Environmental care^3^

The narrowest notion of medicine, according to Nordenfelt, is only concerned with (a), i.e., with “the traditional core activity of the doctor who tries to cure diseases and tries to alleviate the consequences of diseases through palliative measures” [25]. However, a broader understanding of medicine will encompass parts of some of the other areas mentioned and I would argue that, in addition to medical care, at least also some aspects of rehabilitation, medical prevention, and health education are integral parts of what it means to be a physician.

Going back to the thought experiment, if one has a narrow view of medicine (i.e., only (a)), I think it would be harder to argue against the use of the hypothetical safe sleeping pill compared to if one has a broader view of medicine. Why? Well, if medicine is defined in such a narrow way, and if hence the means of the physician are narrowed to “the manipulation of the person’s body or mind through medication, surgery or therapy” [25], then what rationale is there to withhold the prescription of something that is not dangerous and can make the patient feel better?^4^ But if, on the other hand, the goals of medicine also encompass health *education* (f), things may be seen differently. If the role of the physician is not only to prescribe or operate, but also to educate, well, then it would seem odd to first educate the patient on the relationship between existential distress and insomnia, and then to prescribe something that bypasses the problem that has just been identified. Hence, there seems to be something in health education that sometimes can counterbalance the urge to prescribe and that can perhaps even sometimes lead the physician to what has been called “caring deliberation” [5]. According to this view, it is part of the physician’s role to sometimes act more like a teacher (c.f. medicine as “health education” according to Nordenfelt). Going back to the thought experiment, a hypothetical future Toring might *demand* the perfect sleeping pill but, from a health education point of view, it is conceivable that a future physician would refuse to prescribe it, not on grounds of safety, but because of a “broader” view of what medicine is about – a view that includes education and a refusal to narrow down medicine to only pills and surgery.^5^

Discussing existential and relational issues with patients and their effect on health and disease can be seen as not only permissible but as an integral part of what it means to be a physician – at least from a health education point of view. Toring contemplates the broad issues of the “meaning of life” – and this kind of issues should, in my opinion, be a perfectly legitimate topic of discussion with patients. Needless to say, the physician should beware of using his position of power to promote his own worldview, but gently educating the patient about how existential distress might impact sleep is, from that perspective, not only permissible but is part and parcel of what it means to be a health care professional.

Of course, it is possible to argue against such a “broad” view of medicine. It is possible to stick to a narrow view and even to combine this with a consumerist view – the physician being seen as a technical expert that delivers solutions to a health-customer [5]. Medicine may perhaps in the future develop in such a direction; the goals of the medical profession are not set in stone. And that is precisely why it is important to discuss issues such as the ones raised in the present paper. As the thought experiment makes clear, pharmacological treatments are a central part of the practice of medicine, which leads us to the concept of *pharmaceuticalisation*.

## The pharmaceuticalisation of life?

Medicalisation is a well-known concept in medical sociology, and the pharmaceutical industry has been said to have a growing role in medicalisation [29]. The related but distinct notion of pharmaceuticalisation is defined in Table 2. Another related concept is human enhancement, or even neuroenhancement, meaning that biomedical technology is used to improve the performance of a human being who otherwise is not in need of a “cure” [15, 21].

**Table 2:** Some key aspects of pharmaceuticalisation as formulated by Williams and co-workers [29]

Pharmaceuticalisation…	… is a complex, heterogenous socio-technical process involving the discovery, development, commercialization, use and governance of pharmaceutical products centred around chemistry-based technology
	… is about the translation or transformation of human conditions, capabilities and capacities into opportunities for pharmaceutical intervention
	… may extend beyond the strictly medical to the non-medical (i.e., to “healthy” people)
	… entails a network of institutions, organisations, actors and artefacts, as well as the cognitive structures associated with the creation, production and use of new therapeutics
	… is centred on the chemistry-based technology embodied in the pill

Two empirical studies in the USA suggest that the period 1993-2007 was characterized by a process of medicalisation of sleeplessness [24], but that this was followed by an opposing trend of *de*-medicalisation during 2008-15 [23]; the authors speculate that the latter was influenced by, among other things, the increasingly negative portrayals of sedative-hypnotics medicines. Hence, medicalisation and pharmaceuticalisation are dynamic and potentially bi-directional processes [29]. In a UK context, it seems unclear if sleep problems are being subject to a process of pharmaceuticalisation or if, on the contrary, a degree of depharmaceuticalisation will occur in the future (c.f., nonpharmacological treatments such as Cognitive Behaviour Therapy for Insomnia – CBTI) [8, 13, 16].

According to Williams et al, even in cases when drugs are withdrawn from the market, it is more likely that one class of drug will replace another than that drugs will stop being used altogether [29]. The historical shift depicted in Fig. 3 is congruent with such a view; barbiturates gave way to bensodiazepines, which were followed by Z-drugs. Although it seems sensible to think that danger and side-effects are drivers of depharmaceuticalisation, the appearance of a new and safer class of drugs would oppose depharmaceuticalisation – one class of drugs would simply replace the other. Perhaps one could then speak of a process of *re*-pharmaceuticalisation (from one class of drug to another).

To date, it seems clear that the drawbacks of sleeping pills still act like a brake on the pharmaceuticalisation of sleep. And this takes us back to the thought experiment which is central to this paper. What if, in the future, this brake disappears? Will there be any arguments left against the pharmaceuticalisation of sleep? I contend that there are such medico-philosophical arguments. For instance, as was mentioned in the previous section, defining the goals of medicine is important in this respect; if the goals of medicine include education, this can act like a brake against pharmaceuticalisation. But even if one, for the sake of argument, sticks to a narrow view of the goals of medicine as described above, I contend that there are interesting and solid arguments that speak against such a process of pharmaceuticalisation. For instance, when debating the issue of human enhancement and the question of our alleged wish to be “better than well” [11, 15], I think the worry Elliot puts into words has the potential to move the discussion forward [7]:

The traditional worry about enhancement technologies is that users of the technologies are buying individual well-being at the expense of some larger social good. I may improve my own athletic ability by taking steroids, but I set off a steroid arms race that destroys my sport. I may get cosmetic surgery for my “Asian eyes” or use skin lighteners for my dark skin, but I reinforce the implicitly racist social norms that say that Asian eyes or dark skin are traits to be ashamed of. The worry is that some aspect of the way we live together, collectively, is going to be damaged by actions that we take individually […] My worry is that we will ignore important human needs at the expense of frivolous human desires; that dominant social norms will crowd out those of the minority; that the self-improvement agenda will be set not by individuals, but by powerful corporate interests; and that in the pursuit of betterment, we will actually make ourselves worse off.”

Here, Elliot approaches the question of human enhancement from a broad, societal perspective, i.e., not looking only on the right of the individual to act as he/she wants but instead asking “big questions” about what kind of society we desire to live in. Of course, those having a very strict individualistic ethic that absolutizes the principle of autonomy and the rights of the individual might discard this worry as more or less irrelevant. And, needless to say, it is far from self-evident that everything in the quote above is applicable to the question of sleep. Rather, my point is that Elliot helps us to ask alternative questions and to broaden our views concerning this kind of issues. Going back to the question of sleep and the thought experiment: Do we really want to live in a society where the consequences of existential and relational distress are more often than not “fixed by a pill”? Would that not amount to the pharmaceuticalisation of life itself? Would that be desirable?

## Data Availability

N.A.

## Footnotes

1 What happens to him afterwards, in the second part of the book, will be the subject of a future paper.

2 An anonymous reviewer helped me realize that I have *assumed* that the understanding of insomnia as a risk factor for other medical conditions would not have changed dramatically 100 years from now. Hence, I have assumed that the assessment of “risk” would continue to be done on group level, with relatively small effect sizes. But what if, in the future, science enables us to measure the biology of medically dangerous insomnia on the individual level? A putative biomarker could show this for a particular patient (“precision medicine”). Then, the thought experiment would be not only about the safety of sleeping pills, but also about a *tradeoff* (for each patient) between the medical risk of not prescribing and the (inexistant) pharmacological risk of prescribing. That would indeed influence the discussion. However, and conversely, there would presumably also be a group of patients whom would be shown *not* to be at medical risk from insomnia. For them, the thought experiment is not only still valid, but all the more so because the physician would know that no medical harm would befall the patient if sleeping pills were not prescribed; the only argument for prescribing would the subjective discomfort of the patient.

3 a-d are together labeled as “health care”, whereas e-h are called “health promotion”

4 Of course, as we will see in the next section, there might be such a rationale even if one sticks to a narrow view of medicine, but my point here is simply that such arguments would in that case have to be brought in from outside the field of medicine itself, i.e., they would have to belong to the field of philosophy of medicine, not to medicine itself.

5 An anonymous reviewer wondered if there is a crucial and unstated assumption in my work, namely, that I hold as an ideal of human existence to live as free of pharmaceuticals as possible. I do not think that I have such a strong ideal. When people seek the help of their doctor for a given problem, the ideal (in my opinion) is not to keep them as free of medicines as possible, but to offer the “best” possible answer to their request. As a practicing physician, I’m not a “pharmacological minimalist” who in every situation tries as much as possible to avoid prescribing. But I do think physicians need not only *episteme* and *techne*; they also need *phronesis* [6]. And *phronesis* (finding the “best” course of action towards attaining a goal) is dependent on a perceived *telos* for medicine – which is why I introduced Nordenfelt’s thoughts. If education is part of the goals of medicine, then *phronesis* implies an ability to identify when to prescribe and when to educate.

## References

[1] Boyd KM (2000). Disease, illness, sickness, health, healing and wholeness: exploring some elusive concepts. Med Humanit.

[2] Brülde B (2001). The goals of medicine. Towards a unified theory. Health Care Anal.

[3] Burman D (2017). Sleep Disorders: Insomnia. FP Essent.

[4] Buysse DJ (2013). Insomnia. JAMA.

[5] Bäckryd E (2016). "Professional Helper" or "Helping Professional?" The Patient-Physician Relationship in the Chronic Pain Setting, With Special Reference to the Current Opioid Debate. J Contin Educ Health Prof.

[6] Bäckryd E (2019). Nurturing the virtues: Upholding professionalism in the midst of busy medical practice. J Contin Educ Health Prof.

[7] Caplan A, Elliott C (2004). Is it ethical to use enhancement technologies to make us better than well?. PLoS Med.

[8] Coveney C, Williams SJ, Gabe J (2019). Medicalisation, pharmaceuticalisation, or both? Exploring the medical management of sleeplessness as insomnia. Sociol Health Illn.

[9] Ekirch AR (2015). The modernization of western sleep: or, does insomnia have a history. Past Present.

[10] Ekirch AR (2018). What sleep research can learn from history. Sleep Health.

[11] Elliott C (2004). Better than well: American medicine meets the American dream.

[12] Encyclopaedia Britannica. Vilhelm Moberg. https://www.britannica.com/biography/Vilhelm-Moberg. Accessed 9 Sept 2018.

[13] Gabe J, Coveney CM, Williams SJ (2016). Prescriptions and proscriptions: moralising sleep medicines. Sociol Health Illn.

[14] Glatt MM (1962). The abuse of barbiturates in the United Kingdom.

[15] Hall W (2004). Feeling 'better than well'. EMBO Rep.

[16] Haynes J, Talbert M, Fox S, Close E (2018). Cognitive Behavioral Therapy in the Treatment of Insomnia. South Med J.

[17] Hofmann B, Solomon M, Simon JR, Kincaid H (2017). Disease, Illness, and Sickness. The Routledge Companion to Philosophy of Medicine.

[18] Levenson JC, Kay DB, Buysse DJ (2015). The pathophysiology of insomnia. Chest.

[19] Lopez-Munoz F, Ucha-Udabe R, Alamo C (2005). The history of barbiturates a century after their clinical introduction. Neuropsychiatr Dis Treat.

[20] Marinker M (1975). Why make people patients?. J Med Ethics.

[21] Maturo A (2012). Medicalization: current concept and future directions in a bionic society. Mens Sana Monogr.

[22] Moberg V (1953). Sömnlös.

[23] Moloney ME, Ciciurkaite G, Brown RL (2019). The medicalization of sleeplessness: Results of U.S. office visit outcomes, 2008-2015. SSM Popul Health.

[24] Moloney ME, Konrad TR, Zimmer CR (2011). The medicalization of sleeplessness: a public health concern. Am J Public Health.

[25] Nordenfelt L (2001). On the goals of medicine, health enhancement and social welfare. Health Care Anal.

[26] Siriwardena AN, Qureshi MZ, Dyas JV, Middleton H, Orner R (2008). Magic bullets for insomnia? Patients' use and experiences of newer (Z drugs) versus older (benzodiazepine) hypnotics for sleep problems in primary care. Br J Gen Pract.

[27] Swedish board of health and welfare. Statistikdatabas för läkemedel. http://www.socialstyrelsen.se/statistik/statistikdatabas/lakemedel. Accessed 27 Aug 2018.

[28] WHO Collaborating Centre for Drug Statistics Methodology. ATC//DDD Index 2018. https://www.whocc.no/atc_ddd_index/. Accessed 7 Sept 2018.

[29] Williams SJ, Martin P, Gabe J (2011). The pharmaceuticalisation of society? A framework for analysis. Sociol Health Illn.

[30] Williams SJ, Wolf-Meyer M. Longing for sleep: assessing the place of sleep in the 21st century. – Part I, II and III. Somatosphere. 2013; Available at: www.somatosphere.net/. Accessed 29 Nov 2019.

[31] Winokur A (2015). The Relationship Between Sleep Disturbances and Psychiatric Disorders: Introduction and Overview. Psychiatr Clin North Am.

